# Rats and mice rapidly update timed behaviors

**DOI:** 10.1007/s10071-025-01930-9

**Published:** 2025-01-24

**Authors:** N. Aggadi, S. Krikawa, T. A. Paine, P. Simen, C. D. Howard

**Affiliations:** Neuroscience Department, Oberlin College, 173 Lorain St, Oberlin, OH USA

**Keywords:** Interval timing, Learning, Dynamic timing

## Abstract

Keeping track of time intervals is a crucial aspect of behavior and cognition. Many theoretical models of how the brain times behavior make predictions for steady-state performance of well-learned intervals, but the rate of learning intervals in these models varies greatly, ranging from one-shot learning to learning over thousands of trials. Here, we explored how quickly rats and mice adapt to changes in interval durations using a serial fixed-interval task. In the first experiment, animals experienced randomly selected fixed-intervals of 12, 24, 36, 48, or 60 s, for blocks ranging from 13 to 21 trials. Consistent with previous work, animals abruptly increased lever pressing as reward availability approached, and these ‘start times’ scaled with the interval duration for both species. We then quantified the rate of updating to new trial durations and found that rodents consistently updated their start times within 2–3 trials following a change in interval duration, before stabilizing their behavior by the third or fourth trial. To account for repeated exposures to fixed-interval durations, a second set of animals was tested with new fixed-intervals after being trained on the serial fixed-interval task described above. Next, a third group was trained on fixed-interval durations that were generated *de novo* in each day. In each of these contexts, rodents rapidly increased or decreased their start times to mirror new FI durations following exposure to 1–2 trials of new intervals following block transitions. This work adds to growing evidence for rapid duration learning across species, highlighting the need for timing models to be capable of rapid updating in dynamic temporal scenarios.

**Supplementary Information:**

The online version contains supplementary material available at 10.1007/s10071-025-01930-9.

## Introduction

Timing is a crucial aspect of survival, and appropriate behaviors require rapid updating to mirror changes in a dynamic temporal environment. For example, honeybees adjust their foraging schedules to mirror nectar availability in flowers, and they are capable of continually updating this schedule throughout the season based on changes in the environment to optimize their effort (Moore 1989). Bees additionally measure how long it takes to unload their cargo following foraging and decide wether or not to perform a dance to indicate the location of food (Seeley et al. 1992). Optimal foraging strategy requires animals to continually update their expectation of reward timing to determine how to best use their time (Brunner et al. 1992). Timing is also pivotal in social behaviors and reproductive strategies. Males of many species of songbird, for instance, modify second-to-second timing of their elaborate courtship songs based on what they experience in their environment. This precise timing increases their chances of attracting mates and successfully reproducing (Williams 2017).

Studies investigating interval timing, the processing of time intervals ranging from seconds to minutes, have a rich history of using fixed-interval durations to measure timing. However, studies measuring adaptation of behavior following changes in interval duration are relatively rare, although previous work has shown that both rodents and humans can rapidly alter behavior to mirror changes in time expectation. Li and Dudman (2013) demonstrated that mice alter their wait times to mirror delay durations, often adjusting their behavior after only a few exposures to new delay periods. Staddon and colleagues showed similar findings in pigeons (Innis and Staddon 1971; Ludvig and Staddon 2004), though rodents may not adapt symmetrically to increases and decreases in time intervals (Lejeune et al. 1997).

Highlighting the speed of this process, a recent study demonstrated that mice adjust response rates immediately following a single exposure to a distinct trial duration (Xie et al. 2023). Rats share this sensitivity, as they are capable of scaling lever pressing to match changes in fixed-interval durations, seemingly after only a few uncued exposures to changes in duration (Mello et al. 2015). This rapidly learned timing usually follows multiple days of training on aspects of these tasks. Remarkably though, rodents appear capable of learning to time lever pressing within a single behavioral session, despite never performing a timing task previously (Reyes et al. 2020).

How rodents learn to optimize behaviors to mirror a dynamic temporal environment is unknown, but two recent studies have provided strong evidence that rats and mice rely on temporal error monitoring (Akdoğan et al. 2017), a form of temporal metacognition, to estimate their timing accuracy. Indeed, both species are capable of accurately estimating their error in timing and relying on this information to optimize timed behaviors (Kononowicz et al. 2022; Öztel and Balcı 2024). Humans are also capable of this rapid updating and sensitivity to changes in timed durations, even without using counting strategies. In a beat-the-clock task in which participants must withhold responses as long as possible but without going over a time limit to maximize reward, subjects could optimize responses after 1–2 trials following a change in target time (Simen et al. 2011). Thus, several species of mammals and birds appear capable of rapid updating of behavior to changes in anticipated time durations, though few studies have quantified the rate of this adaptation.

Rate of learning is also a key distinguishing feature between many models of interval timing. For example, one-shot learning rates are achievable in both Drift Diffusion Models (DDM; Rivest and Bengio 2011; Simen et al. 2011) and pacemaker accumulator models (Gibbon et al. 1984; Treisman 1963; see Simen et al. 2013 for review). These models posit the continuous accumulation of a quantity, such as action potentials, over time to make timing decisions. Classic pacemaker–accumulator models (Creelman 1962; Treisman 1963) count pulses emitted at a fixed rate; different durations lead to different pulse counts. Related models such as the Time-adaptive opponent Poisson DDM (TopDDM) (Balci and Simen 2016) and the Behavioral Theory of Timing (BeT; Killeen and Fetterman 1988) instead time different durations by adapting the rate of accumulation up to a fixed threshold. Either approach enables organisms to rapidly adjust their timing behaviors in response to changing temporal contexts. In contrast to these approaches, some use iterative learning algorithms based on machine learning techniques to learn over a much longer time scale (Buonomano 2005) and Long Short-Term Memory mechanisms (Hochreiter and Schmidhuber 1997). These more general machine learning approaches require many more exposures to a new duration to learn the necessary adjustments of neural network weights to time new durations (Rivest and Bengio 2011).

The aim of this study is to characterize the rate of learning of new intervals in rodent models performing a standard fixed-interval (FI) timing task. To do so, we rely on a ‘serial fixed-interval’ (sFI) task previously used with rats (Mello et al. 2015), in which rewards are delivered if animals press a lever after a fixed-interval of time (12–60 s) in blocks of 13–21 trials. Following the completion of each block, a new interval is presented, and subjects have to update their responses accordingly. To determine the flexibility of responding and updating following interval changes, we assessed four metrics of estimated start times of periods of high-rate responding: the first press in each trial, the time of first burst (three subsequent presses in the shortest 40% of inter-press intervals), the time when press rate raised twofold above baseline responding, and change points detected using a Bayesian algorithm (Balci et al. 2009). This approach builds on a rich history of single-trial analysis previously used in rodent (Church et al. 1994) and pigeon (Cheng and Westwood 1993) timing tasks.

## Materials and methods

### Animals

All experiments were approved by the Oberlin College Institutional Animal Care and Use Committee and were conducted in accordance with the National Institutes of Health’s Guide for the Care and Use of Laboratory Animals and Animal Research: Reporting of In Vivo Experiments (ARRIVE) guidelines. Rats and mice were maintained on a 12 h/12 hr light/dark cycle and were provided *ad libitum* access to water. Experiments were carried out during the light cycle using a total of 40 male Sprague Dawley rats (Hilltop Laboratories) 60–75 days old at the start of the study and 46 C57BL/6J (Jax Strain #: 000664) male and female mice 60–90 days old at the start of the study. To support motivated responding during sFI training, rats and mice were maintained at 85% free-feeding weight, and rats were allowed to gain ~ 5 g per week to mirror their normal growth curve.

### **Serial fixed**-**interval training**

Rats and mice were trained on a serial fixed-interval task developed by Mello et al. (2015). Training took place in standard Med-Associates operant behavioral chambers for rats or mice, which were outfitted with a central food magazine and retractable levers on either side. Operant chambers had a central houselight on the wall opposite the food magazine, and 45 mg or 20 mg dustless precision pellets were used in rat and mice experiments, respectively (F0021 for rats, F0071 for mice; Bio-serv). Rodents were initially trained using one day of magazine training. Here, sessions began with the simultaneous illumination of the houselight and delivery of pellets every 30 s until 60 rewards were earned.

During the following 3–4 days, rodents were trained in continuous reinforcement (CRF) to press the left lever for pellets using a fixed ratio 1 schedule, where one press yielded one reward. This task began with the illumination of the houselight and extension of the left lever. Rats and mice could earn 75 or 50 rewards maximum, respectively, and training continued until 30 min elapsed or the maximum number of rewards were earned.

**Fig. 1:**
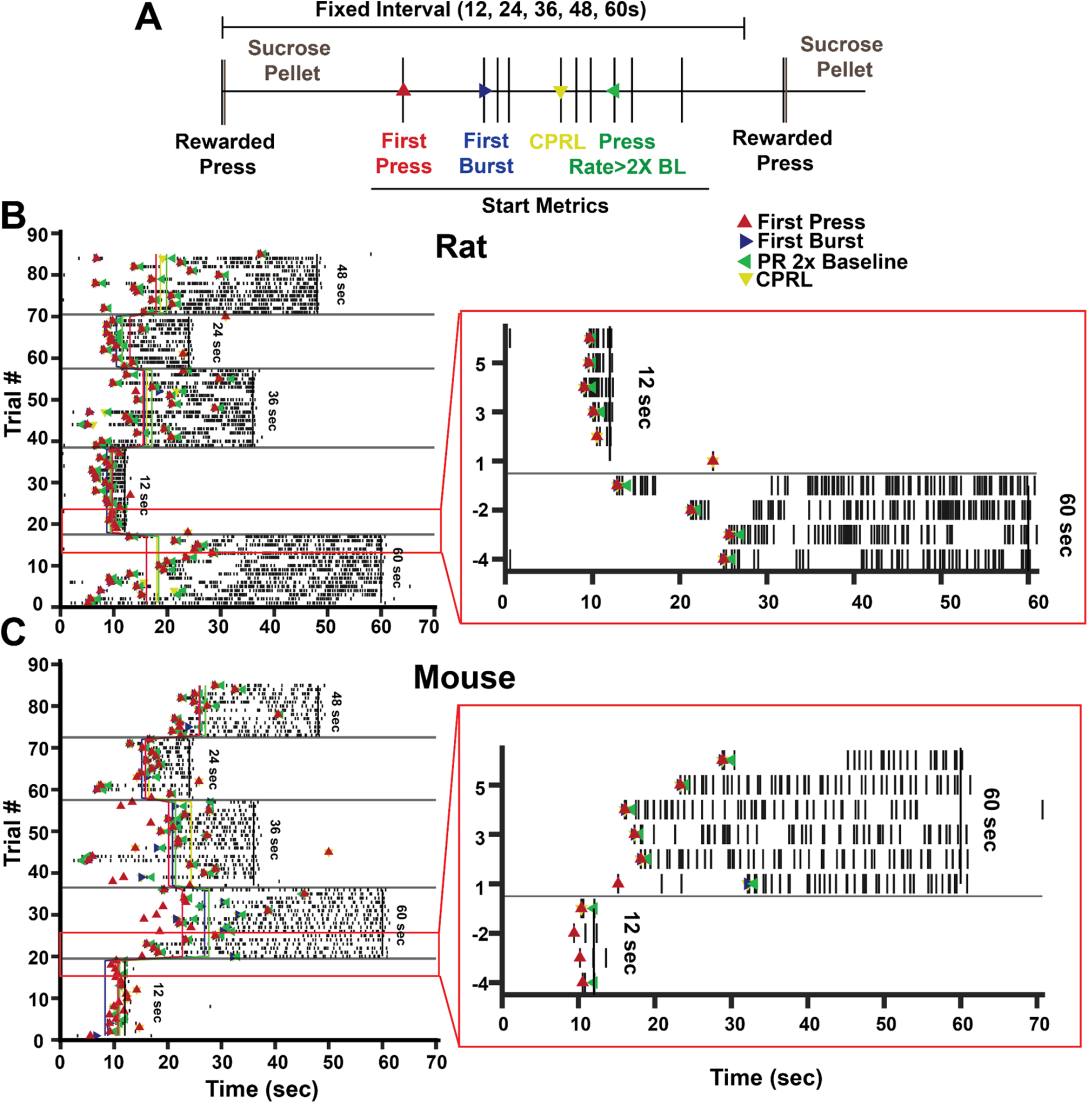
Experimental design and representative pressing data. **A.** Both rats and mice were trained on a serial fixed-interval task (sFI) where FI trials of 12, 24, 36, 48, or 60 s were drawn randomly without replacement and presented for blocks of 13–21 trials. Pressing in each trial was assessed to determine the first press, first burst (three presses in a row within the fastest 40% of all inter-press intervals for each experiment), the time when press rates doubled over baseline responding of each experiment, and change points detected using a Bayesian change point detection algorithm (CPRL; Balci et al. 2009). Each start-point metric was determined independently and could occur at the same press, but they are shown here separated for clarity. At the end of each block, the FI duration changed, and no cues were presented to alert rodents to block transitions. Representative rat (**B**) and mouse (**C**) pressing behavior in the sFI task. First press (red), first burst (blue), press rate increase (green), and CPRL (yellow) are shown trial by trial as dots, blockwise averages are shown as vertical lines of corresponding color, the deadline of each block is shown as a vertical black line, and each press is shown as a small vertical dash in each trial. Block transitions are shown as horizontal gray lines. Presses earlier than 5 s in each trial were excluded from analyses and are not shown in the raster plot. The insets (right) show 10 trials around the first block transitions zoomed in for clarity

Following the completion of CRF training, rodents began training on the serial fixed-interval task (sFI). This task began with the illumination of the houselight and extension of the left lever. Fixed-interval (FI) durations were drawn without replacement from a list of 12, 24, 36, 48, and 60 s and were presented in blocks of 13, 15, 17, 19, or 21 trials, which was also drawn randomly from a list without replacement. If all five possible values were used, both lists were refreshed before a randomly drawn value was repeated, and lists were refreshed at the beginning of each training session. Rodents could freely press the lever during the FI, and the first press after the end of the FI resulted in delivery of a pellet and the beginning of the next trial (Fig. 1A). Importantly, only the delivery of the pellet signaled the end of the trial. No other cues signaled transitions between trials or between blocks. Rodents continued sFI training each day until 100 rewards were earned or until 90 min had elapsed. Behavioral training occurred daily for mice and every weekday for rats. Analysis was conducted on the 20th day of training for both species.

In Experiment 2, a subset of these same rats (*n* = 11) and a new group of mice (*n* = 9) were trained on this sFI task before being tested on an sFI task with trial durations they had never seen before. This task was identical to the sFI task described above with the exception of FI durations, which were 13, 15, 31, 45, and 57 s. These values were randomly generated in Matlab and represent the first randomly generated value that falls within 60–140% of each of the original FI values. This test was performed to determine how animals respond to intervals that they had never experienced during training.

Finally, a new group of rats (*n* = 12) and mice (*n* = 9) were trained on a sFI task identical in design to the initial sFI task, but with FI durations that were generated *de novo* each day. Here, the initial list of FI values (12, 24, 36, 48, and 60 s) was randomly drawn from without replacement and multiplied by (0.8 + X*(0.4)), where X is a random value between 0 and 1. This resulted in a range of values 80–120% of the original FI values. This was done to ensure rodents saw a distinct trial duration each day, but also to ensure that FI durations did not largely overlap. After 20 days of training on this task, rodents were then presented with the original sFI task, where performance was assessed. This test was done to ensure rodents did not simply memorize FI durations learned during training, which may result in seemingly rapid updating of behavior when FI duration changes.

### Data analysis

Analysis was performed using custom-written analysis scripts in Matlab (2020a, Mathworks). Each trial was assessed for four start time estimates: the time of the first press in each trial, the first burst in each trial, the time the press rate elevated above 2× baseline press rate, and the first detected Bayesian change point (CPRL; Balci et al. 2009). Importantly, as all trials ended in rewards and pressing often extended beyond reward delivery, start times were only assessed 5 s or later into each trial. Examples of early presses are shown in Fig. 1B and C. Though it was possible for animals to experience six or more blocks in a session, analysis was restricted to the first five blocks as responding diminished in later blocks.

**Fig. 2:**
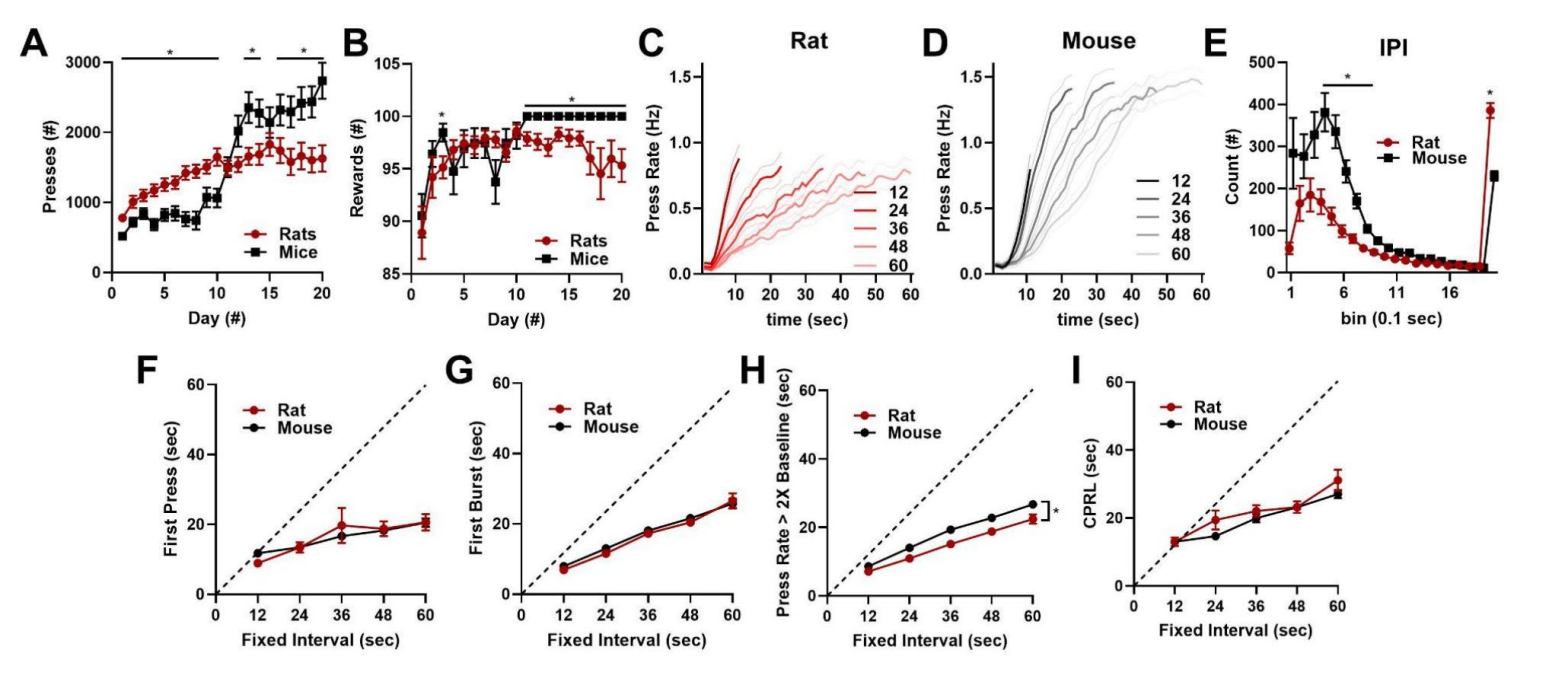
Task acquisition and scaling of pressing behaviors to FI duration. **A.** Number of presses for both rats and mice across training. **B**. Number of rewards earned by rats and mice across training. **C–D**. Press rates for each FI duration at day 20 of training for rats (**C**) and mice (**D**). **E**. Inter-press interval distribution for each species. **F–I**. Start time estimate averages shown by FI duration for first press (**F**), first burst, (**G)**, press rate increase (**H**), and change point (**I**; CPRL). *indicates significant *post hoc* Tukey test for **A** and **B** and Šidák test for **E**. * beside bracket indicates significant effect of species in **H**

A ‘burst’ was defined as three presses separated by inter-press intervals that fell within the smallest 40% of inter-press intervals for each experiment. Previous studies have used inter-response intervals to determine the beginning of “bouts” of behavior. However, we opted to define brief periods of high pressing as bursts, as bouts tend to describe a period of longer engagement that might have multiple bursts within (Shull et al. 2001). Time of press rate elevation was calculated by determining the press rate in each second of a trial, then smoothing these values using a moving average with a window size of three sec. Next, the average press rate was determined across the entire session, and trials were assessed to find the time when the smoothed press rate went above 2× this average. Thus, burst and press rate start times took into account the differences in baseline pressing across individuals, which accounted for some differences in how rats and mice engaged in the task (Fig. 2E). Finally, trial-by-trial change points were determined based on a previously described Bayesian change point algorithm (CPRL; Balci et al. 2009) with a relative likelihood (Bayes Factor) of 10. This analysis works by assessing each interpress interval and determining how well each subsequent data point fits a change vs. no-change model and assigning it a likelihood factor. With this approach there can be many change points detected in a data set. We therefore defined CPRL change points as being the first point in each trial with a likelihood factor of 10 or higher.

**Fig. 3:**
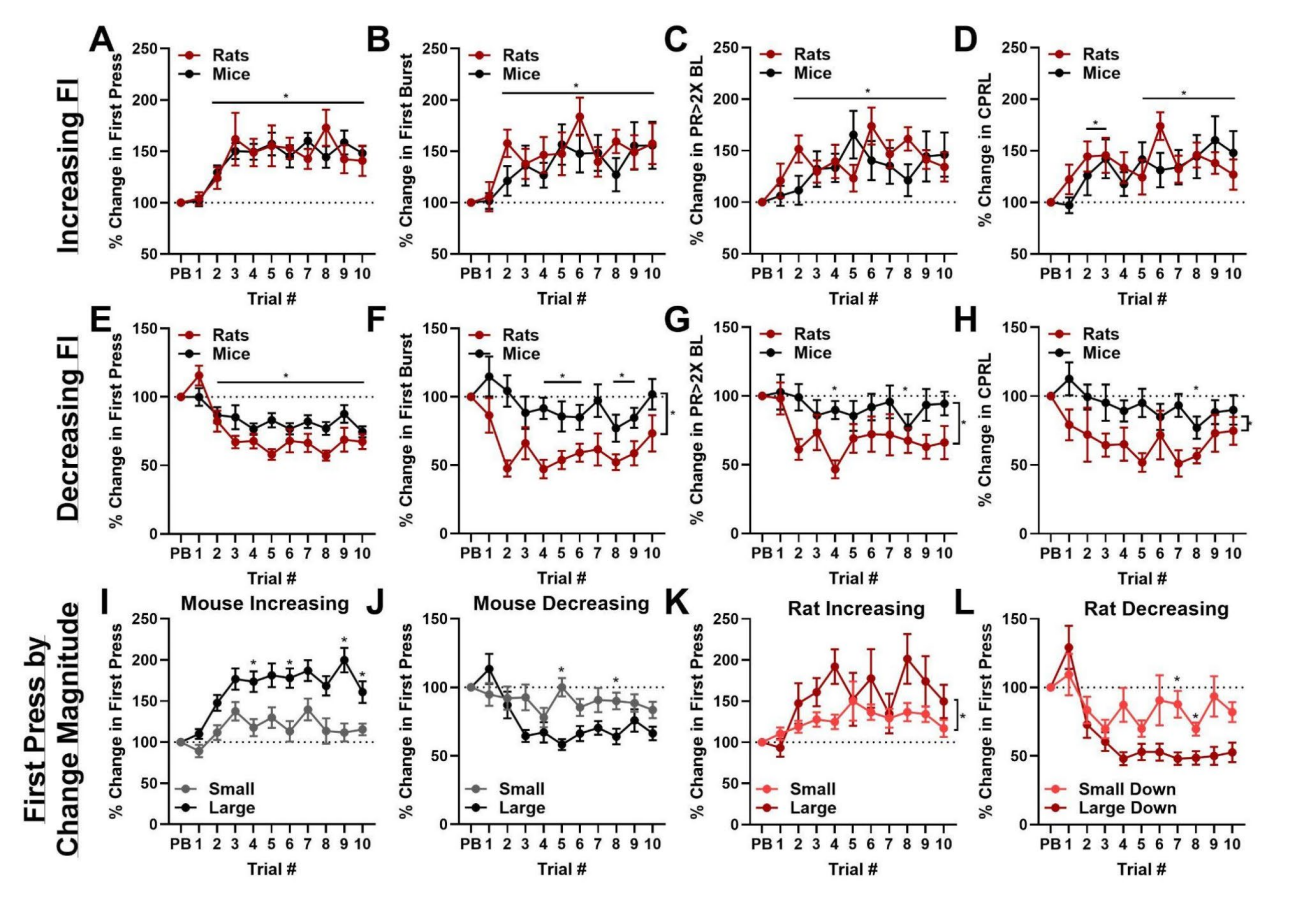
Changes in start time estimates across FI block transitions. **A–D.** Start time estimates following increasing block transitions normalized to previous block (PB) average for first press (**A**), first burst (**B**), time when press rate surpassed 2 × baseline (**C**; PR > 2X BL), and change point estimate (**D**). **E–H.** Start time estimates following decreasing block transitions normalized to previous block (PB) average for first press (**E**), first burst (**F**), time when press rate surpassed 2 × baseline (**G**; PR > 2X BL), and change point estimate (**H**; CPRL). **I–L.** First press times grouped by small or large magnitude FI duration changes. Increasing transitions are shown in **I** for mice and **K** for rats, and decreasing transitions are shown in **J** for mice and **L** for rats. *indicates significant *post hoc* Dunnett’s or Sidak’s multiple comparisons test vs. PB in **A–H** or against large vs. small magnitude response times for **I–L**. * beside bracket indicates significant effect of species or magnitude in **F–H** or **K**

**Fig. 4:**
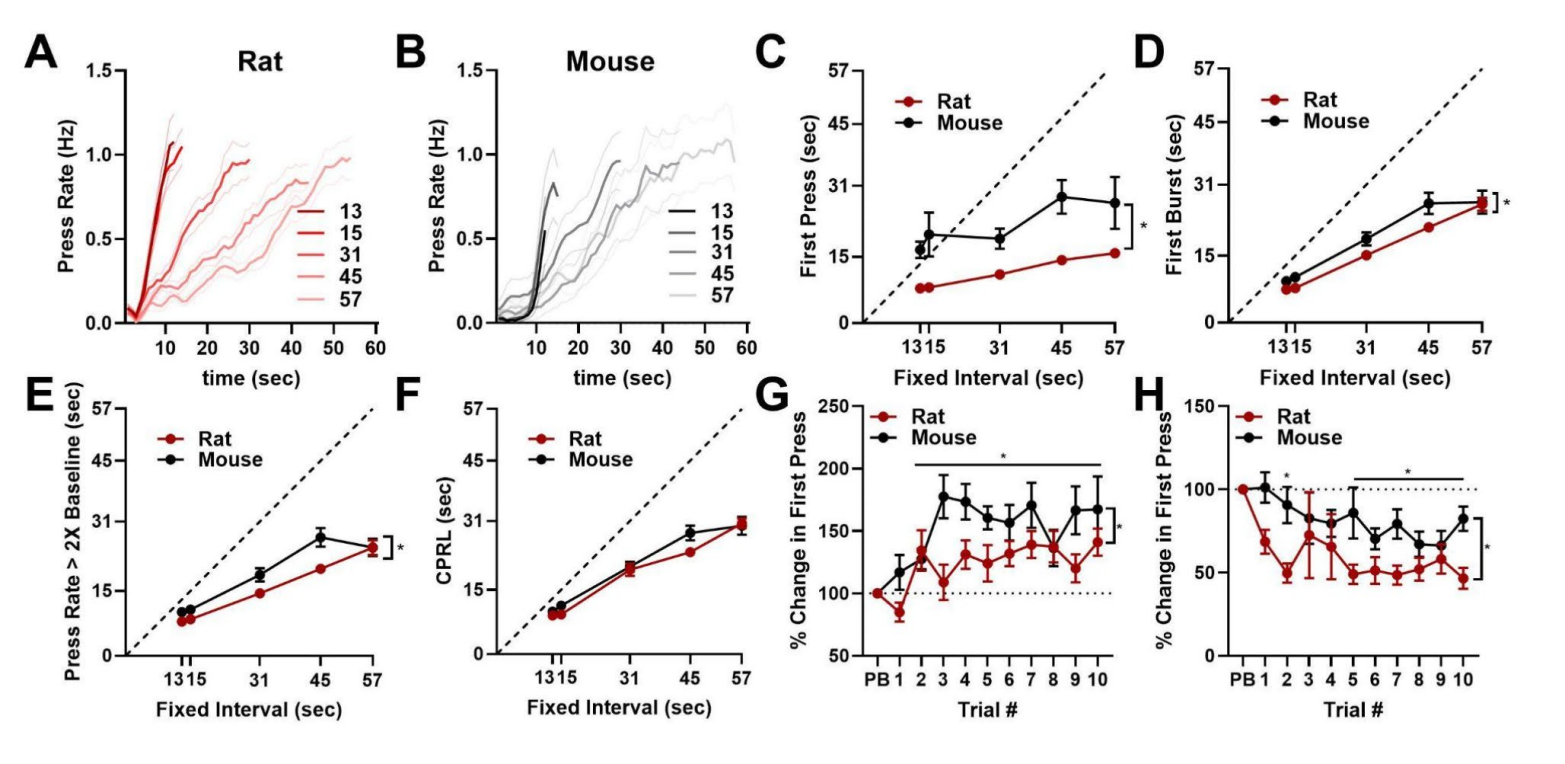
Performance of rats and mice on a sFI task with novel durations. **A–B.** Press rates for each FI duration for rats (**A**) and mice (**B**). Start time estimate averages shown by FI duration for first press (**C**), first burst (**D**), press rate increase (**E**), and change point (**F**; CPRL). **G–H**. First press normalized to previous block (PB) average for increasing (**G**) and decreasing block transitions (**H**). *indicates significant *post hoc* Dunnett’t multiple comparison test vs. PB in **G–H**. * beside bracket indicates significant effect of species in **C–E** and **G–H**

**Fig. 5:**
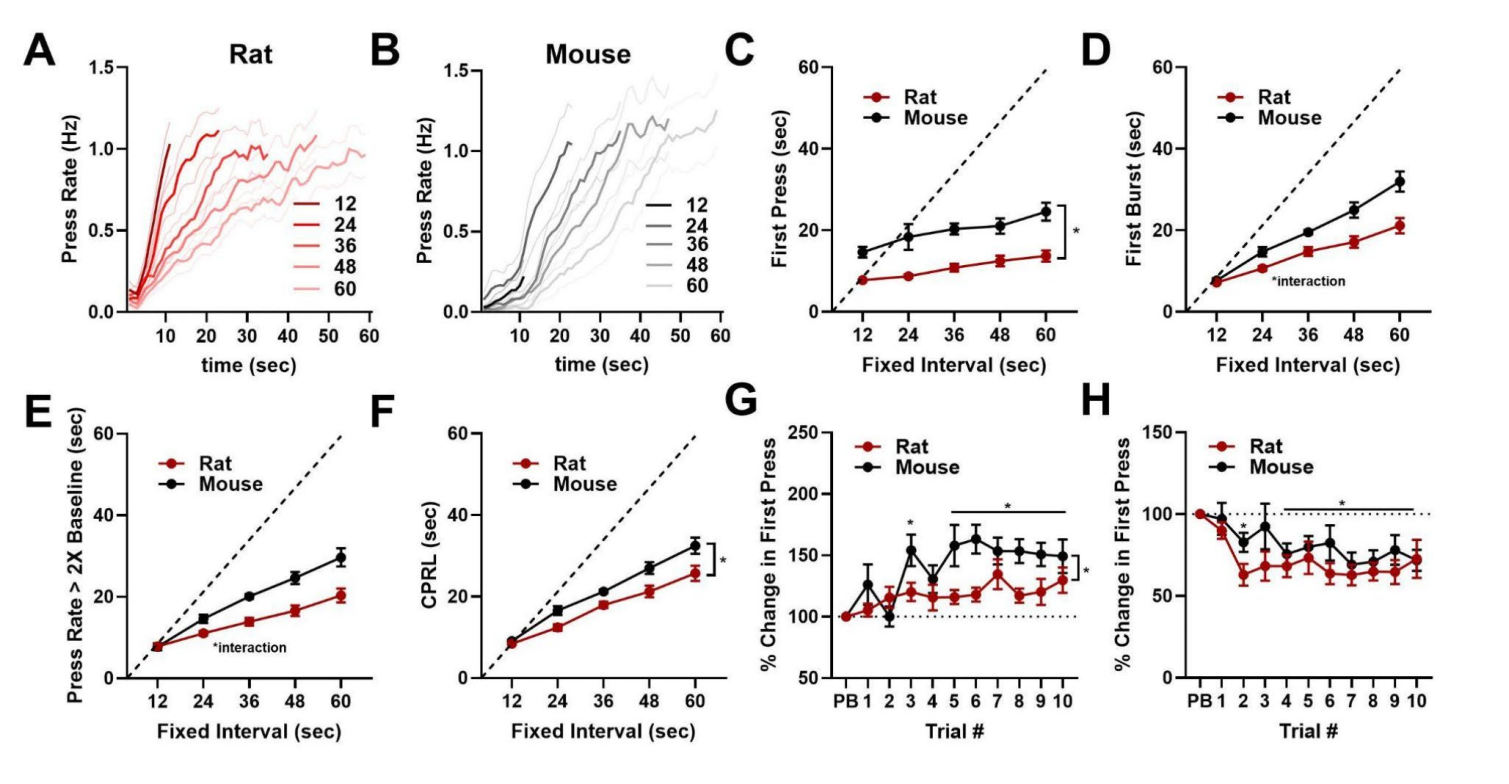
Performance of mice on the sFI task after being trained on unique intervals generated each day. **A–B**. Press rates for each FI duration for rats (**A**) and mice (**B**). Start time estimate averages shown by FI duration for first press (**C**), first burst (**D**), press rate increase (**E**), and change point (**F**; CPRL). **G–H**. First press normalized to previous block (PB) average for increasing (**G**) and decreasing block transitions (**H**). *indicates significant *post hoc* Dunnett’t multiple comparison test vs. PB in **G–H**. * beside bracket indicates significant effect of species in **C**,** F**, and **G**

As the order of FI durations was randomly determined each day, it was not possible to assess particular transitions (e.g. 12 to 60 s) with robust statistical power. However, these individual transitions are shown in Supplemental Figure S1. Therefore, we collapsed transitions into increasing and decreasing transitions to assess rates of updating following FI change (Figs. 3, 4G–H and 5G–H). Here, the previous block-wise average start time was compared to subsequent trials following the transition. Mice had particularly reduced press rates in short duration (12 s) FI trials (see Figs. 2D, 4B and 5B), which made accurate assessment of start times as assessed by first burst, press rate increase, and CPRL impossible. Therefore rate of updating for these metrics was restricted to transitions between durations of 24, 36, 48, 60 s and block transitions to or from 12 s were excluded. All block transitions were included for assessment of first press. To assess how the magnitude of FI duration changes impacted responding, we also grouped data by ‘small’ and ‘large’ transitions (Fig. 3I–L). Here, small transitions were defined as being between FI durations that are directly adjacent (for example, 36 to 48 s). Large transitions were defined as being between FI durations separated by at least two potential FI durations (for example, 60 s to either 24–12 s).

### Statistical analysis

Species comparisons were performed using a Greenhouse-Geisser corrected (when there is a lack of sphericity) two-way repeated-measures ANOVA (Figs. 2A, B, E and F, 3A, E, I and J, 4C, G and H and 5C and H; Supplemental Figures S4A, B, C, D, E, G) or two-way repeated-measures mixed model (Figs. 2G, H and I, 3B, C, D, F, G, H, K and L, 4D, E and F and 5D, E, F and G; Supplemental Figures S4 F, H) in the case of missing values with *post hoc* Tukey, Šidák, or Dunnett’s tests. Trial-by-trial correlations of FI duration and first press were performed using Pearson’s correlations (Supplemental Figure S2–3). Data analysis was performed using custom-written scripts in MATLAB (2020a, Mathworks). All statistics and graphical output were conducted using Prism 10 (GraphPad).

## Results

### Experiment 1: Serial fixed-interval performance

Rats (*n* = 28) and mice (*n* = 24) were trained on a serial fixed-interval task (sFI) where fixed-interval trials of 12, 24, 36, 48, or 60 s were presented in blocks of 13–21 trials before the interval changed (Fig. 1A). We assessed pressing behavior using four start time metrics: (1) the first press in each trial, (2) the first burst (3 successive presses within the fastest 40% of inter-press intervals in each experiment), (3) the time when press rates reached twice the baseline response rate and (4) the time when a significant change point was detected based on a Bayesian approach (CPRL; Balci et al. 2009). Representative behavior on day 20 of sFI training shows that rats (Fig. 1B) and mice (Fig. 1C) are capable of scaling start times to mirror new FI durations by waiting longer to press on longer FI trials.

We assessed performance on the sFI task at day 20 to determine if rats and mice had learned to adjust start times based on FI duration. Both species increased pressing across training, though mice increased pressing more at the end of training (significant species × day interaction, F_(19, 931)_ = 18.32, *p* < 0.0001; Fig. 2A). Accordingly, both rats and mice increased rewards earned during training, with mice earning more at the end of training (significant species × day interaction, F_(19, 950)_ = 2.167, *p* = 0.0027; Fig. 2B). Consistent with previous work (Mello et al. 2015), rats and mice increased press rates earlier on shorter FI durations (Fig. 2C–D), though mice tended to press very little on 12 s trials. Overall, mice pressed at a significantly higher frequency, and rats had more long inter-press intervals (significant species × bin interaction, F_(19, 950)_ = 9.584, *p* < 0.0001; Fig. 2E). Both rats and mice scaled blockwise average start time estimates to mirror FI durations as assessed by first press (significant effect of fixed-interval, F_(1.79, 89.32)_ = 10.24, *p* = 0.0002; Fig. 2F), first burst (significant effect of fixed-interval, F_(1.70, 83.65)_ = 152.8; *p* < 0.0001; Fig. 2G), press rate increase (significant effect of fixed-interval, F_(2.14, 106.7)_ = 223.1, *p* < 0.0001; Fig. 2H), and change point (significant effect of fixed-interval, F_(2.37, 99.09)_ = 145.8, *p* < 0.0001; Fig. 2I), with mice increasing press rates later than rats (significant effect of species, F_(1, 50)_ = 18.41, *p* < 0.0001; Fig. 2H). In sum, rats and mice both learn to optimize responding in a dynamic timing task by scaling lever press start times to mirror FI durations.

We next sought to characterize the rate at which start time estimates shifted following block transitions to assess how quickly rodents could adapt to changes in FI durations. Because block transitions were not consistent across experiments (FI duration was randomly drawn without replacement), it was not possible to assess specific block transitions (i.e. isolate only 36 to 60 s transitions) with robust statistical power (these transitions are shown in Supplemental Figure S1). Therefore, we opted to collapse all decreasing and increasing block transitions to measure the general rate of adaptation. Furthermore, we found that many animals had a high portion of single presses just after the 12 s FI deadline, which resulted in few bursts, press rate changes, and change points in 12 s blocks. Thus, we further narrowed our assessment to transitions between 24, 36, 48, and 60 s blocks for these later three start time estimates.

To measure the rate of adaptation to new fixed-intervals, we determined the block average of each start time estimate from the block preceding a transition and compared that to subsequent trials. When the previous block transitioned to a longer FI duration, rats and mice significantly delayed their first presses (significant effect of trial, F_(5.24, 261.8)_ = 7.079, *p* < 0.0001; Fig. 3A), first bursts (significant effect of trial, F_(6.52, 190.3)_ = 4.705, *p* < 0.0001; Fig. 3B), press rate increases (significant species × trial interaction, F_(10, 296)_ = 1.920, *p* = 0.042; Fig. 3C), and change points (significant effect of trial, F_(6.67, 180.8)_ = 3.710, *p* = 0.0011; Fig. 3D). Similarly, when the previous block transitioned to a shorter FI duration, rats and mice significantly expedited their first presses (significant species × trial interaction, F_(10, 47)_ = 2.335, *p* = 0.011; Fig. 3E), first bursts (significant effect of trial, F_(5.25, 125.4)_ = 3.730, *p* = 0.003; Fig. 3F), press rate increases (significant effect of trial, F_(6.33, 159.4)_ = 2.297, *p* = 0.035; Fig. 3G), and change points (significant effect of trial, F_(5.87, 121.5)_ = 2.598, *p* = 0.022; Fig. 3H). Rats and mice were largely consistent in rate of updating start estimates, though rats sped up responding more during decreasing FI durations relative to mice when assessed by first burst (significant effect of species, F_(1, 28)_ = 14.20, *p* < 0.001; Fig. 3F), press rate increases (significant effect of species, F_(1, 28)_ = 4.913, *p* = 0.035; Fig. 3G), and change point estimates (significant effect of species, F_(1, 28)_ = 7.665, *p* < 0.01; Fig. 3H). We then used *post hoc* Dunnett’s tests to assess when start time estimates significantly deviated from baseline and found that rats and mice were capable of altering responses by the second trial after transitions for all start time estimates (*p* < 0.05; Fig. 3A–D). On the other hand, rats and mice were generally slower at adapting to decreasing FI durations (significant Dunnett’s test on trial 4, 4, and 8 for first burst, press rate increase, and change point, respectively; Fig. 3F–H), though rats and mice significantly altered their first press times by the second trial after block transition (Fig. 3E). In summary, rats and mice both rapidly adapted to changes in FI duration, with both species significantly altering at least one start time estimate following a single exposure to a new FI duration.

Previous work noted that when rats transition from a learned FI to a longer or shorter FI in a peak interval task (an FI task with a number of “probe” trials in which no reward is delivered), peak responding times tended to shift from the original FI to the geometric mean between these two intervals (Meck et al. 1984). Here, rodents appear to display intermediate start times between the previous block average and start time estimates that occur later in the block (e.g. Figure 3A, presses at trial 2 occur at ~ 125% before settling at ~ 150%). Therefore, we analyzed each unique transition type (e.g. 12 to 48 s) to determine if consistent intermediate responses occurred following FI transitions (Supplemental Fig. 1). Average start time estimates do not appear to consistently take place between previous block averages and responses later in the block. Rather, responding seems to jump following transitions suddenly before returning closer to previous block averages, though quantitative assessment of this data set is not possible due to very few unique transition types. Thus, the intermediate values noted in Fig. 3 likely represent a subset of animals that have immediately updated their response times being averaged with those of a subset that updated their responding later, rather than consistent partial updating in all animals.

We next aimed to explore if changes in start times were consistent for transitions of differing FI-duration change magnitude. We anticipated that animals would more dramatically update their responses when transitioning between FI’s with large time differences (e.g. 12 to 60 s), vs. those with smaller differences in duration (e.g. 24 to 36 s). We therefore grouped transitions based on magnitude of FI duration changes, with adjacent values (e.g. 12 to 24 s) being categorized as ‘small’ and with FI’s separated by at least two possible durations (e.g. 12 to 48 or 60 s) being categorized as ‘large’. During increasing FI transitions, both mice (significant magnitude × trial interaction; F_(10, 380)_ = 2.083, *p* = 0.025; Fig. 3I) and rats (significant effect of magnitude; F_(4.21, 234.3)_ = 3.606, *p* = 0.0062; Fig. 3K) updated start times more when transitions were relatively larger. Similarly, during decreasing transitions, both mice (significant magnitude × trial interaction; F_(10, 360)_ = 2.780, *p* = 0.0025; Fig. 3J) and rats (significant magnitude × trial interaction; F_(10, 35)_ = 2.588, *p* = 0.0049; Fig. 3L) more robustly modified start times during transitions of larger magnitude. Together, these data suggest that rodents in a dynamic timing environment where they have learned that FI durations can change (see Discussion) do not respond consistently at intermediate values while transitioning in favor of sudden changes in time estimates, but they do make bigger adjustments to start times when FI changes are larger.

As rats and mice scaled their average start time to the FI duration (Fig. 2F–I), we next characterized the rate at which this positive correlation develops. In the first trial after a block transition, it is very unlikely performance will scale with FI duration as the animal does not yet know the FI has changed. A positive relationship between start times and FI duration could develop rapidly or slowly over subsequent trials. As expected, rats and mice display no positive correlation between first press time and FI duration in the first trial after transition (Supplemental Figure S2 and 3 A). However, as early as the second trial after transitions for rats or the third trial after transitions for mice, both species develop a significant positive correlation between first press times and FI duration that lasts into the fourth trial after transition (Supplemental Figure S2 and 3 B–D). Across all four measured start time estimates, R^2^ values rise from the first to the second trial after the transition before stabilizing by the fourth trial after block transitions (Supplemental Figure S2 and 3E). Taken together, these results further demonstrate that rodents adapt to changes in FI duration by updating timed responses following a single exposure to a new FI duration and by reaching asymptote by the third or fourth trial.

### Experiment 2: Performance on a serial fixed-interval task with new durations

This rapid updating of timed behaviors could reflect rodents adapting behavior to new FI durations, or it is possible that rodents have over-learned these intervals during training and remember them from previous exposure. To distinguish these different possibilities, we trained a group of rats (*n* = 11) and mice (*n* = 9) on the sFI task described above and then tested them on an sFI task with new FI intervals of 13, 15, 31, 45, and 57 s. Rats and mice largely scaled press rates to mirror FI durations, but both species responded similarly during 13 and 15 s durations (Fig. 4A–B). Both species also scaled average start times in each block with these FI durations for first press (significant effect of fixed-interval, F_(2.30, 41.39)_ = 11.00, *p* < 0.0001; Fig. 4C), first burst (significant effect of fixed-interval, F_(2.33, 39.08)_ = 93.27, *p* < 0.0001; Fig. 4D), press rate increase (significant effect of fixed-interval, F_(2.43, 43.10)_ = 86.09, *p* < 0.0001; Fig. 4E), and CPRL (significant effect of fixed-interval, F_(2.71, 35.86)_ = 112.2, *p* < 0.0001; Fig. 4F), though mice tended to wait longer to make first presses (significant effect of species, F _(1, 18)_ = 14.19, *p* = 0.0014; Fig. 4C), first bursts (significant effect of species, F_(1, 18)_ = 5.368, *p* = 0.0325; Fig. 4D), and to increase press rate (significant effect of species, F_(1, 18)_ = 9.518, *p* = 0.0064; Fig. 4E).

We next assessed the rate of updating following block transitions, omitting burst, press rate, and change point estimates due to a smaller sample size. Rats and mice were again highly sensitive to changes in FI duration, adapting to both increases (significant effect of trial, F_(5.64, 101.5)_ = 4.787, *p* < 0.001; Fig. 4G) and decreases (significant effect of trial, F_(2.45, 44.10)_ = 3.150, *p* = 0.043; Fig. 4H) in FI durations by the second trial after transitions. In this task, mice displayed greater adaptation to increasing FI durations (significant effect of species, F_(1, 18)_ = 9.738, *p* < 0.01; Fig. 4G), while rats displayed greater adaptation to decreasing FI durations (significant effect of species, F_(1, 18)_ = 7.589, *p* = 0.013; Fig. 4H). Thus, both rats and mice rapidly updated their responses to FI durations that they had not previously seen, suggesting previous knowledge of exact FI durations is not required to rapidly update timed responses.

### Experiment 3: Performance on serial fixed-interval after training on new durations each day

While these results suggest prior exposure to FI durations is not required for rapid adaptation to new FI durations, it remains possible that repeated training on a fixed set of trial durations may result in animals “categorizing” these new FI durations into 12-, 24-, 36-, 48-, or 60-sec-like durations. Thus, we conducted a final experiment where a new subset of mice (*n* = 9) and rats (*n* = 12) were trained using FI durations that were generated *de novo* each day. To ensure that FI durations were not clustered together too much (e.g. 12, 11.9, and 12.1 s), we generated FI durations by randomly scaling 12, 24, 36, 48, 60 s by a factor ranging from 80 to 120%. Rodents were trained using this modified sFI task for 20 days, and on the 21st day, they were tested with the original sFI task using 12, 24, 36, 48, 60 s blocks in a random arrangement, which they then saw for the first time.

Rats and mice again increased press rates differently across FI blocks by waiting longer to escalate pressing on longer FI durations (Fig. 5A–B). Both species scaled average start times based on FI durations, though mice tended to wait longer on all trials before making first presses (significant effect of fixed-interval, F_(2.905, 55.19)_ = 18.64, *p* < 0.0001; significant effect of species, F_(1, 19)_ = 27.90, *p* < 0.0001; Fig. 5C), first burst (significant fixed-interval × species interaction, F_(4, 70)_ = 4.071, *p* = 0.005; Fig. 5D), increasing press rate (significant fixed-interval × species interaction, F_(4, 71)_ = 4.222, *p* = 0.004; Fig. 5E), and change points (significant effect of fixed-interval, F_(2.58, 40.70)_ = 77.64, *p* < 0.0001; significant effect of species, F_(1, 19)_ = 9.056, *p* = 0.007; Fig. 5F). Despite training on different FI durations each day, rats and mice both significantly altered first presses during increasing (significant effect of time, F_(6.43, 121.4)_ = 4.888, *p* < 0.0001; Fig. 5G) and decreasing FI durations (significant effect of time, F_(5.01, 95.21)_ = 5.260, *p* < 0.001; Fig. 5H) by at least the third trial following increasing or decreasing block transitions (significant Dunnett’s test, Fig. 5G–H). Mice adapted more robustly than rats following increases in FI in this task (significant effect of species, F_(1, 19)_ = 17.35, *p* < 0.001; Fig. 5G). In sum, rodents are capable of rapidly updating timed responses when trained on novel FI durations each day, suggesting this rapid updating is not simply due to storing previously learned FI durations and treating new intervals as falling into previously learned groupings.

We next explored if rats and mice differed in their rate of adapting to new interval duration increases vs. decreases within species. To do this, we rearranged first press data from experiments 1–3 to directly compare responding during increasing and decreasing block transitions (Figure S4). Rats and mice across all experiments consistently differ in how they respond to increases and decreases in interval (significant trial × direction interaction; all *p* < 0.05; Supplemental Fig. 4A–F). Dunnett’s multiple comparison test was used to determine the trial that first statistically differed from the previous block average. Consistently, in Experiments 1–3, rats updated responding to decreasing interval durations more rapidly than during increasing interval durations (Supplemental Fig. 4A, C, and E). In contrast, mice in experiments 1–3 all consistently updated responding more rapidly during increases in interval durations (Supplemental Fig. 4B, D, and F). However, it is possible that this finding is impacted by relatively low sample size. To explore this possibility, increase the robustness of the statistical analysis, and explore the generalizability of these findings across all three experiments, we combined the results from experiment 1–3 within species. In this context, rats and mice both significantly altered their start times by the second trial following a block transition for both increasing and decreasing transitions (Supplemental Fig. 4G and H). Overall, this result suggests that both species are similarly fast in updating their responses to changes in interval duration and that this updating does not largely differ between increasing or decreasing block durations within species.

## Discussion

### Summary of findings

This study sought to compare rates of adapting to changes in temporal environments between two species commonly used for interval timing studies: rats and mice. Overall, we find that mice and rats are highly flexible in a dynamic timing task, and that they can rapidly update timed responses to changes in FI durations, often only 1–2 trials after experiencing a change in interval duration. Both species had similar adaptation rates, though they were somewhat different in their sensitivity to increases vs. decreases in duration. Additionally, mice tended to press at higher frequencies overall and waited longer to begin pressing in all three experiments (but see Limitations below). Together, this work provides a novel assessment of inter-species learning rates in a dynamic timing task, finding that rodents are highly capable of robust and near-immediate behavioral adaptation to changing time intervals.

### Rapid updating of timed responses across species

We found that both rats and mice learned to adapt their time estimates completely within 3 exposures to a new duration in a serial fixed-interval task. By some metrics, they showed evidence of learning following a single exposure to a new FI duration. This evidence for rapid duration learning in rodents bolsters existing evidence that humans (Simen et al. 2011), rodents (Li and Dudman 2013; Mello et al. 2015; Xie et al. 2023) and birds (Innis and Staddon 1971; Ludvig and Staddon 2004) can learn from 1 to 2 exposures to a new duration. In rhesus monkeys trained on a FI task, a short or long interval duration resulted in underestimation or overestimation of the following trial, respectively, again suggesting single-trial updating of learned responses (Mendez et al. 2011). Thus, strong evidence for near-immediate updating of timed behaviors has been demonstrated across a wide swath of mammalian and bird species. Similar findings may be found in invertebrate species and organisms with simpler nervous systems, and future studies should explore this possibility (Bosivert and Sherry 2006; but see Craig et al. 2014). This similarity in findings across species suggests a common neural mechanism may support this rapid updating.

We note here that animals do not consistently respond at values between the new FI and old FI values, as has been previously reported (Meck et al. 1984), instead rapidly modifying their start times across each possible transition (Figure S1). This difference can likely be explained by two important differences in task design. First, Meck et al. (1984) used a peak interval design with probe trials to determine peak responding, while here, animals only ever experienced rewarded FI trials. Thus, animals in the current work do not experience probe trials, which likely leads to less uncertainty about new trial times. Second, rodents in this work have been previously trained to anticipate changes in the time intervals, while rats in Meck et al. 1984 experienced changes in interval duration for the first time after being trained on a single interval. Thus rodents here have been trained not only to attune to time, but to attune to changes in time, undoubtedly making them more flexible in their timed responses.

Rodents scaled their average start times to mirror FI intervals (Fig. 2F–I and Figs. 4 and 5C–F). Start times depend linearly on FI duration, with a slope around 0.5. These results mirror human performance in Beat the Clock (Simen et al. 2011), where the slope was closer to 0.9, and a TopDDM model that set its response threshold at 90% of its maximum height accounted for the data. Here, the same model matches the data with a threshold at around 50% of its maximum height. There is an additional complication in the shortest 12-second interval that could reflect rodents taking relatively longer to eat reinforcers on short-duration trials. It is also possible that premature responses on long trials reflect rodents sampling the lever in anticipation of FI block changes. In this task, there is no disincentive to sample the lever early, unlike tasks like Beat the Clock (Simen et al. 2011) where earlier than optimal responding decreases rewards earned. In peak interval tasks, one would expect peak times to better scale with the learned interval (e.g. Church et al. 1994; Toda et al. 2017; Xie et al. 2023).

### Brain mechanisms of temporal adaptation

The neural mechanisms responsible for modifying expectation of reward timing remain largely unknown. It is well-established that neuronal responses scale with FI durations (Wang et al. 2018; Mello et al. 2015), though the brain mechanisms setting the rate of this scaling remain elusive (see De Corte et al. 2022 for review). Xie and colleagues (2023) recently demonstrated that inhibition of anterior lateral motor cortex in M2 drove earlier-than-expected timed licking responses, demonstrating that modifying brain activity in an area involved in motor timing could disrupt timing in future trials. As brain circuits of timing may be intrinsic to brain areas controlling specific aspects of behavior (Paton and Buanomono 2018), it is possible that manipulating areas associated with other timed behaviors will similarly drive anticipatory alterations in timed responses.

In addition, due to its role in reinforcement learning (Schultz 1997), the neurotransmitter dopamine is a candidate molecule for modulating learning about reward timing. Indeed, dopamine responses scale with behavioral timing (Soares et al. 2016) and optogentically modifying dopamine release alters timed responses (Soares et al. 2016; Howard et al. 2017). Similarly, systemic administration of dopamine antagonists (Meck 1986; Buhusi 2002) or direct intracranial infusions into the striatum (De Corte 2019) drive shifts in timed responses, suggesting a crucial role of the nigrostriatal dopamine pathway in modifying the timing of learned behaviors.

Future studies exploring brain activity associated with learning about time are needed. Since animals’ time estimates can update rapidly, any neural mechanism that underlies this learning should display abrupt state change as new intervals are experienced. Further, perturbations of the brain that impact the speed of duration learning will help identify brain areas or cellular processes involved in duration-memory encoding, storage, and retrieval. Parallel work exploring rate-setting elements in models of interval timing and learning will also inform these future studies and help generate testable hypotheses.

### Models of interval timing and temporal learning

The current study quantified rates of updating timed behaviors and strongly suggests rodents learn new durations rapidly — in as little as a single exposure to a new duration, therefore supporting models of timing with fast learning rates. Classic pacemaker–accumulator (PA) models of timing (Creelman 1962; Gibbon et al. 1984; Treisman 1963) predict one-shot learning by having the accumulator essentially acting as noisy stopwatches: counting clock pulses and storing counts in a computer-like memory. Many subsequent models of interval timing have since attempted to achieve greater neural plausibility than digital-circuit-inspired PA models, but these newer approaches are sometimes incapable of one-shot learning of durations. Even simple modifications to the PA framework lead to questions about how, and about how quickly, the resulting models learn. For example, in models where final pulse count totals remain constant and instead the rate of pulses changes for different durations (cf. Killeen and Fetterman 1988), a learning rule is necessary for how to adjust the rate of pulses. Such rules exist (Rivest and Bengio 2011; Simen et al. 2011), and, as in the classic PA models, they predict that learning can take as little as one exposure to a new duration if sufficient information is presented to a subject.

In contrast, models that use more general, iterative learning algorithms, such as stochastic gradient descent, require many training trials (Buonomano and Mauk 1994; Matell and Meck 2004). Models that learn through iterative adjustment of weights, such as gradual adjustment underlying deep learning, require significantly more trials to learn durations relative to rates of learning in the current study (Rivest et al. 2010). Thus, the rate of updating timed responses should serve as a litmus test for determining whether a given timing model is behaviorally plausible.

### Limitations

There are several limitations of the current work. First, while mice tended to wait longer to begin pressing across all three experiments. This may be due to the body size-to-reinforcer ratio differing between mice and rats: mice may have taken longer to eat a relatively larger sucrose pellet. This is best evidenced by the greatly reduced press frequency mice displayed in 12-sec trials, which may be caused by mice eating while the interval elapsed. This feature of the behavioral task obscured our ability to measure alternative estimates of start times beyond first presses. Nevertheless, we opted to include 12-sec trials as pilot studies demonstrated worse scaling of start times to fixed-interval durations when these short trials were excluded.

Next, while we performed multiple experiments to account for the generalization of timed responses and categorization of intervals, it is impossible to discount the notion that rodents responded to new time intervals based on the span of intervals chosen during training. In other words, rodents may have responded to new trials as short-, medium-, or long-like rather than learning a more granular interval (i.e. 57 s). This confound is difficult to resolve, but it is potentially a feature of all timing tasks, as rodents tend to experience previous time intervals throughout their lives prior to training. One previous study suggests that this may not entirely be the case, as prior training using temporal information is not required for animals to learn about time intervals within a single day (Reyes et al. 2020). Additionally, future work could explore more extreme changes in new interval durations, as doubling or halving interval durations (for example) would expand the generalizability of our results and might extract more subtle features of responding during transitioning (Meck et al. 1984; Killeen and Fetterman 1988). Finally, it is possible that scaling of start times to FI durations is a byproduct of longer trials being more likely to provide sufficient time to engage in pressing. Trial-by-trial quantification of scaling (Supplemental Figs. 2 and 3) suggests this is not entirely the case as scaling does not develop until 1–2 trials after transitions.

### Summary

The current work is, to our knowledge, the first study to quantify the rate of learning across species on a dynamic interval task. This approach should be utilized in future studies to explore causative interventions on the rate of learning in addition to steady-state timing. This work supports the notion that mice or rats have similar rates of learning about time to humans and thus similar brain mechanisms of timing may be taking place across species. Finally, this work supports models of timing that reflect rapid rates of updating timed behaviors to changes in interval durations.

## Electronic supplementary material

Below is the link to the electronic supplementary material.

Supplementary Material 1: Fig. 1. Transitions between unique fixed-interval durations. Changes in first presses across block transitions are shown for rats (top) and mice (bottom). Data is grouped by the previous block (12 s, upper left; to 60 s, lower right) and the value of the next block deadline is shown by color (12 s teal; 24 s black; 36 s blue; 48 s red; 60 s green).

Supplementary Material 2: Fig. 2. Correlation of first press times to FI duration following transitions across all rats. **A–D**. First press times are plotted for the first (**A**), second (**B**), third (**C**), and fourth (**D**) trials following a FI transition across all tested durations and rats. The first block in each experiment is excluded from this analysis, and only blocks 2–5 are shown to demonstrate updating from one block to another. **E**. R^2^ values for all four start time estimates are shown across each trial following a block transition. Data were assessed with a Pearson’s correlation.

Supplementary Material 3: Fig. 3. Correlation of first press times to FI duration following transitions across all mice. **A–D**. First press times are plotted for the first (**A**), second (**B**), third (**C**), and fourth (**D**) trials following a FI transition across all tested durations and mice. The first block in each experiment is excluded from this analysis, and only blocks 2–5 are shown to demonstrate updating from one block to another. **E**. R^2^ values for all four start time estimates are shown across each trial following a block transition. Data were assessed with a Pearson’s correlation.

Supplementary Material 4: Fig. 4. Changes in first presses across increasing and decreasing block transitions. Changes in first presses are shown for increasing (red) and decreasing (black) transitions for rats (left) and mice (right). Data from Experiment 1 is shown in A–B, Experiment 2 in C–D, and Experiment 3in E–F. Data collapsed across Experiments 1–3 is shown in G–H. *indicates significant *post hoc* Dunnett’s or Sidak’s multiple comparison test vs. previous block average (PB).

## Data Availability

The datasets generated for this study are available upon request to the corresponding author.

## Abbreviations

ANOVA: Analysis of Variance
sFI: Serial Fixed-Interval
