# Supplementary figures and images for: Rats and mice rapidly update timed behaviors
Source: Anim Cogn. 2025 Jan 24;28(1):6. doi: 10.1007/s10071-025-01930-9 (PMC11759285; doi:10.1007/s10071-025-01930-9)

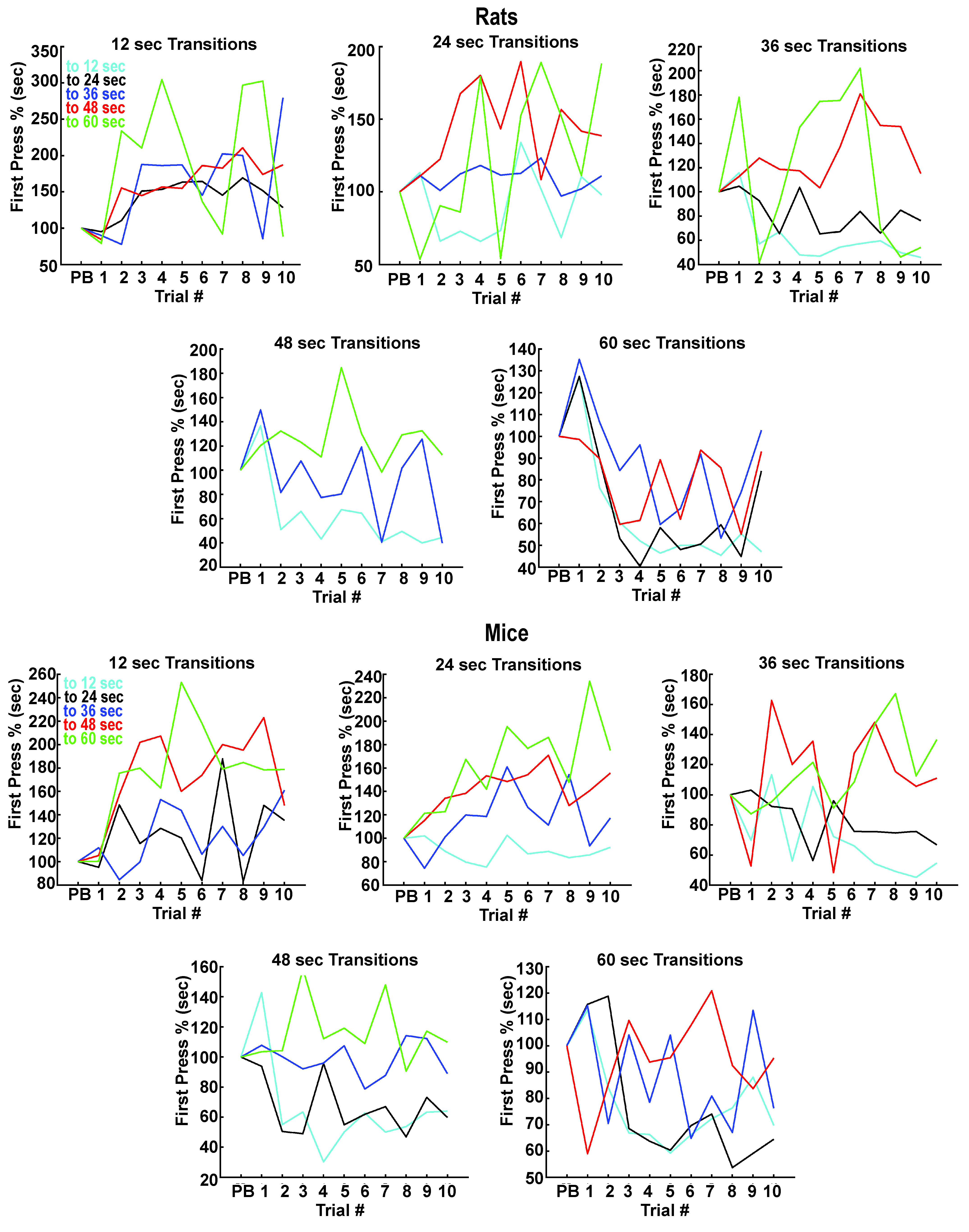

Supplement: Supplementary file 1 — Supplementary Material 1: Fig. 1. Transitions between unique fixed-interval durations. Changes in first presses across block transitions are shown for rats (top) and mice (bottom). Data is grouped by the previous block (12 s, upper left; to 60 s, lower right) and the value of the next block deadline is shown by color (12 s teal; 24 s black; 36 s blue; 48 s red; 60 s green). [file 10071_2025_1930_MOESM1_ESM.jpg]

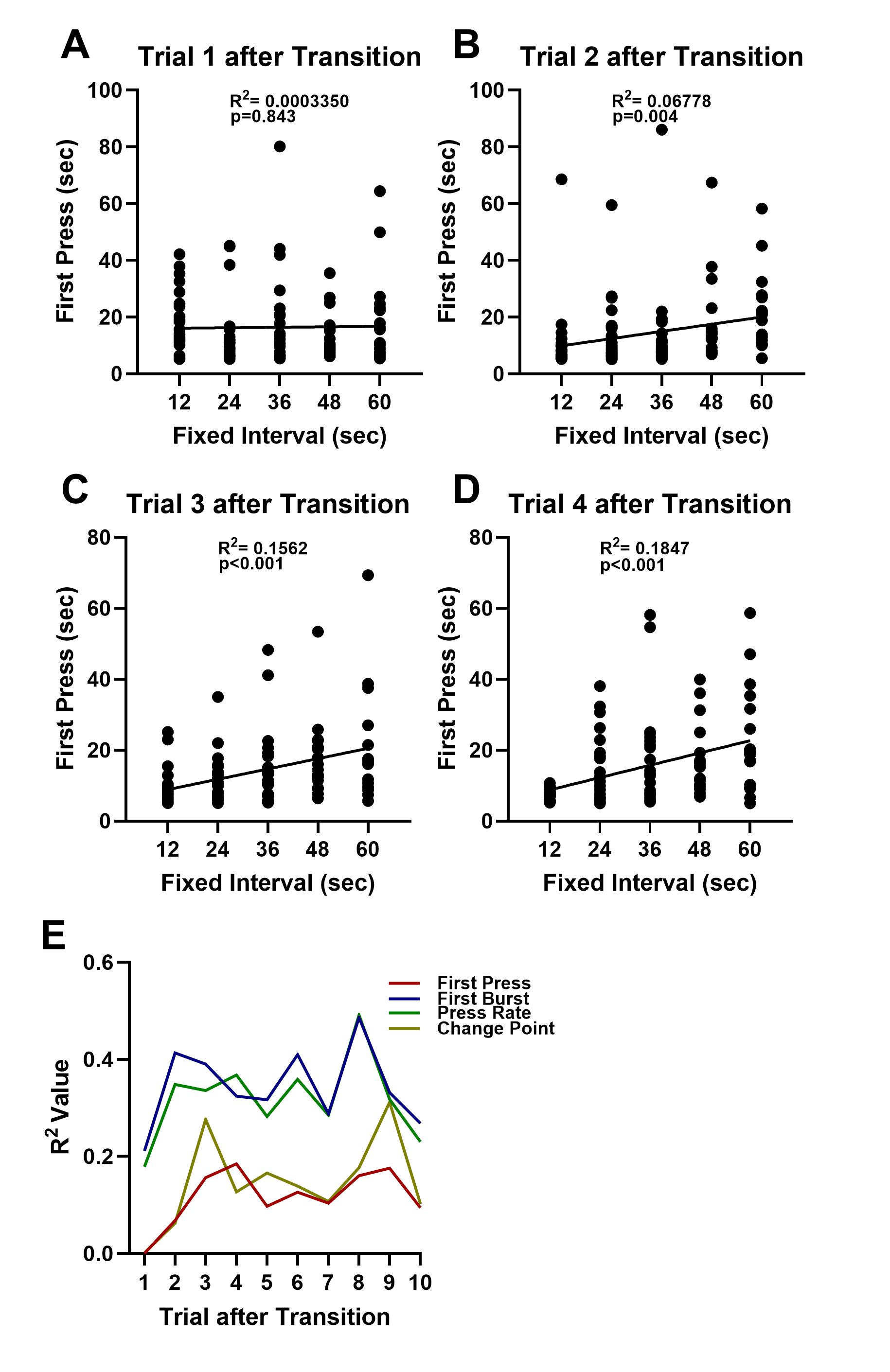

Supplement: Supplementary file 2 — Supplementary Material 2: Fig. 2. Correlation of first press times to FI duration following transitions across all rats. A–D. First press times are plotted for the first (A), second (B), third (C), and fourth (D) trials following a FI transition across all tested durations and rats. The first block in each experiment is excluded from this analysis, and only blocks 2–5 are shown to demonstrate updating from one block to another. E. R2 values for all four start time estimates are shown across each trial following a block transition. Data were assessed with a Pearson’s correlation. [file 10071_2025_1930_MOESM2_ESM.jpg]

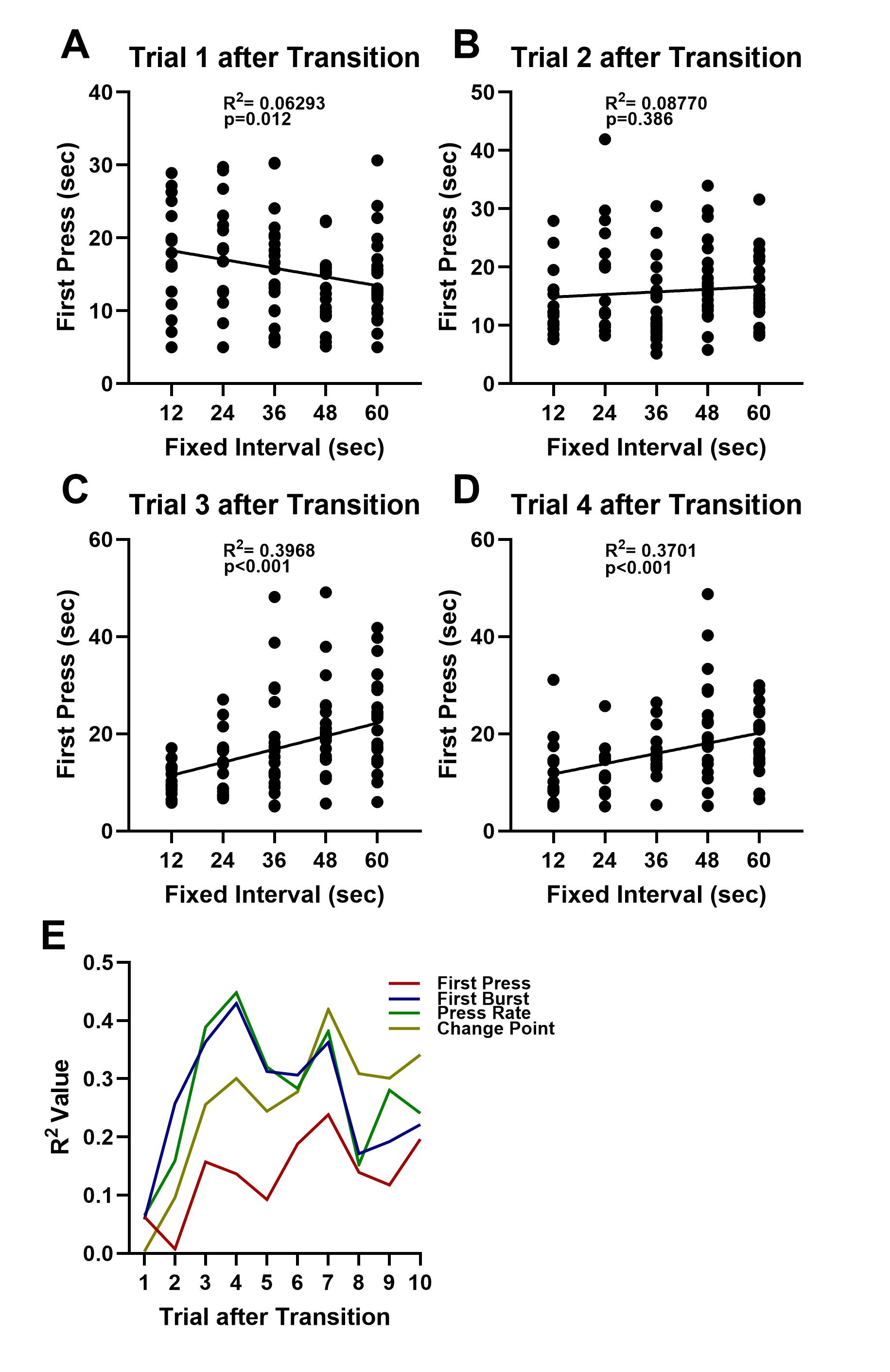

Supplement: Supplementary file 3 — Supplementary Material 3: Fig. 3. Correlation of first press times to FI duration following transitions across all mice. A–D. First press times are plotted for the first (A), second (B), third (C), and fourth (D) trials following a FI transition across all tested durations and mice. The first block in each experiment is excluded from this analysis, and only blocks 2–5 are shown to demonstrate updating from one block to another. E. R2 values for all four start time estimates are shown across each trial following a block transition. Data were assessed with a Pearson’s correlation. [file 10071_2025_1930_MOESM3_ESM.jpg]

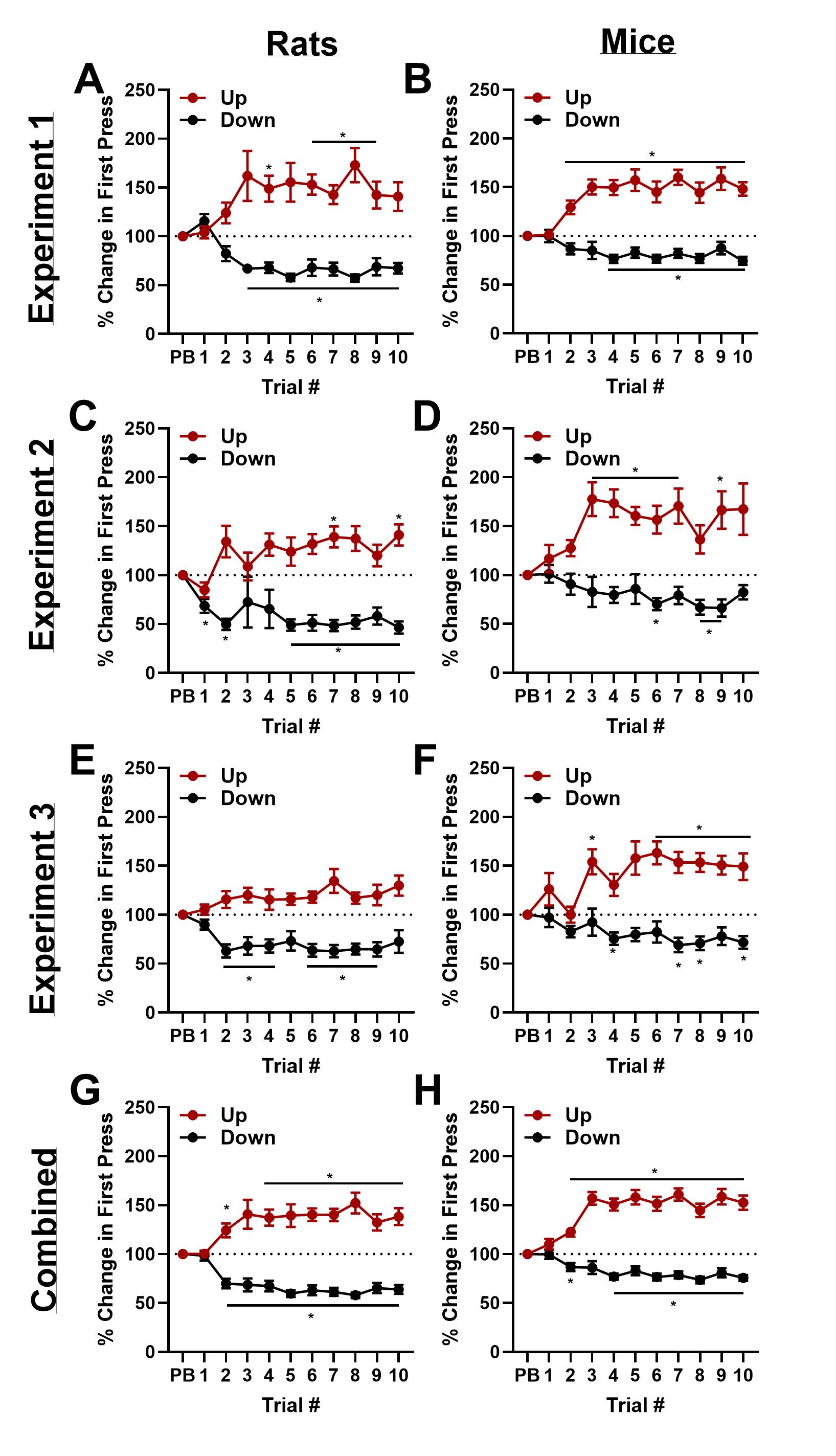

Supplement: Supplementary file 4 — Supplementary Material 4: Fig. 4. Changes in first presses across increasing and decreasing block transitions. Changes in first presses are shown for increasing (red) and decreasing (black) transitions for rats (left) and mice (right). Data from Experiment 1 is shown in A–B, Experiment 2 in C–D, and Experiment 3in E–F. Data collapsed across Experiments 1–3 is shown in G–H. *indicates significant post hoc Dunnett’s or Sidak’s multiple comparison test vs. previous block average (PB). [file 10071_2025_1930_MOESM4_ESM.jpg]
